# Phenotyping first-generation genome editing mutants: a new standard?

**DOI:** 10.1007/s00335-017-9711-x

**Published:** 2017-07-29

**Authors:** Lydia Teboul, Stephen A. Murray, Patrick M. Nolan

**Affiliations:** 1 The Mary Lyon Centre, MRC Harwell Institute, Harwell Science Campus, Didcot, Oxfordshire UK; 2 The Jackson Laboratory, Bar Harbor, ME USA; 3 Mammalian Genetics Unit, MRC Harwell Institute, Harwell Science Campus, Didcot, Oxfordshire UK

## Abstract

The unprecedented efficiency of the CRISPR/Cas9 system in genome engineering has opened the prospect of employing mutant founders for phenotyping cohorts, thus accelerating research projects by circumventing the requirement to generate cohorts using conventional two- or three-generation crosses. However, these first-generation mutants are often genetic mosaics, with a complex and difficult to define genetic make-up. Here, we discuss the potential benefits, challenges and scientific validity of such models.

## Introduction

The Clustered Regularly Interspaced Short Palindromic Repeat/CRISPR associated 9 (CRISPR/Cas9) system is a powerful tool for genome engineering, supporting the rapid production of mutant animals for phenotyping (Wang et al. 2013). Indeed, the first publication showing the use of this RNA-guided endonuclease (RGEN) system in generating insertions/deletions (indels) or point mutations also illustrated the possibility of characterising mutant phenotypes in founder cohorts. Although the original article highlighted the diversity of individuals generated with a given set of single guide RNAs (gRNAs) in any mutagenesis experiment, the publication made no mention of mosaicism nor of rearranged homology-directed repair event in the animals derived from microinjection (F0s). Further studies showed that the CRISPR/Cas9 system can facilitate the generation of even more complex alleles (tagged and conditional alleles) when co-delivered with longer DNA donor sequences (Yang et al. 2013). This seminal work led to proposing RGEN as a means to accelerate genetic studies by generating cohorts of F0 that bear one or multiple mutant alleles, generating either null or point mutations, for phenotyping (Wang et al. 2013).

As use of the technology quickly propagated in biomedical research, it became apparent that a large proportion of F0 animals derived from the microinjection of CRISPR/Cas9 reagents are genetic mosaics, most likely because the RGEN-induced mutagenic events occur following embryo cleavages (Yen et al. 2014; Singh et al. 2015; Mianné et al. 2017). Furthermore, a third of indels in coding regions do not result in a frameshift and therefore, in many cases, do not alter protein function. In a survey of a total of 19 F0 animals from 10 different mutagenesis experiments employing pronuclear microinjection of Cas9 mRNA, sgRNA and donor oligonucleotides, we detected an average of 2.8 alleles per animal, based on PCR amplification and sequencing of genomic DNA extracted from ear biopsies. Adding to the complexity of mosaicism, we and others also described that rearranged alleles (“illegitimate repairs”, Mianné et al. 2016; “KI + indels”; Renaud et al. 2016) are also created in parallel with the correct integration of donor sequences by homology-directed repair (HDR).

Thus, although the CRISPR/Cas9 system can yield very high mutagenesis efficiency, the same process can produce animals with unpredictable and complex genetic make-up that may be difficult to fully unravel. We discuss here the validity of these founders as subjects for phenotypic investigations.

## Mosaic and chimeric analysis

Transient transgenics are unintentional mosaic F0s (Wilkie et al. 1986) and have been very successfully employed to study the regulation of gene expression. Furthermore, genetic mosaics and chimeras are classic experimental subjects that have been employed in both *Drosophila* (reviewed in Perrimon 1998) and mouse (reviewed in Rossant and Spence 1998) genetic studies. Their use enables the dissection of cell autonomous and non-autonomous roles of genes and pathways. A mosaic animal is defined as an organism composed of genetically distinct cells derived from the same zygote, while a chimera, commonly used in mouse studies, is composed of genetically distinct cells from different zygotes, typically an ES cell and embryo donor. Amongst other fields, such paradigms are of great value in developing cancer study models (Wang et al. 2007 and comment by; Lozano and Behringer 2007). Indeed, soon after its description as a genome editing tool, CRISPR/Cas9 was used to generate mosaic tissues in zebrafish liver showing that *Anxa4* is required for liver progenitor viability (Zhang et al. 2014).

However, the complexity of the genetic make-up is quite different in these two classes of model. Chimeric animals consist of two precisely defined cell populations (i.e. through morula aggregation or ES cell injection in blastocyst). In contrast, in animals obtained with CRISPR/Cas9, unpredictable and diverse outcomes arise from mutagenic events from 1-cell onwards through to subsequent cellular cleavages in embryos. In the latter case, each animal is likely to have a unique spectrum of alleles and/or variable contribution of each allele to the whole organism. Moreover, accurate and quantitative ascertainment of the allelic spectrum is challenging because of the diversity of potential outcomes. Given these challenges, we considered whether these new (and more complex) mosaic animals are appropriate models for phenotyping?

## CRISPR/Cas9 F0 phenotyping

Early publications on CRISPR/Cas9-aided in vivo mutagenesis in mouse (Wang et al. 2013) and in zebrafish (Thomas et al. 2014; Ablain et al. 2015) indeed brought a proof to the principle that phenotypes can be observed in cohorts of founder animals. Given the speed and efficiency of CRISPR/Cas9 gene targeting, direct screening of F0 animals could be particularly desirable as it has the potential to reduce time, effort and cost substantially.

### F0 modelling of developmental phenotypes

As mentioned above, anecdotal evidence from early CRISPR experiments indicated that founders could indeed display phenotypes depending on the overall efficiency of the experiment. Additionally, we and others have found that production of KO alleles for genes predicted or known to be embryonic lethal frequently results in fewer than expected live born pups, suggesting lethality of F0 founder animals. Conceivably, therefore, developmental phenotypes could be directly assessed in F0 embryos, providing a potentially rapid means for testing hypotheses. For example, Guimier et al. showed that direct mutagenesis of the mouse orthologue of *MMP21* could recapitulate the left–right asymmetry defects (presenting as heterotaxy) and associated congenital heart defects (CHD) in F0 embryos, validating the discovery of this gene in multiple human CHD pedigrees (Guimier et al. 2015). In this case, the efficiency of mutagenesis and penetrance of phenotypes was quite high, and the authors additionally showed that both KO and KI/KO allelic combinations were obtained and displayed the mutant phenotype despite clear mosaicism in some of the specimens examined. F0 mutagenesis has also been applied to scenarios that are challenging to model using standard mouse breeding. For example, Liu et al. were able to establish the digenic aetiology of the closely linked genes *Sap130* and *Pcdha9* in development of hypoplastic left heart syndrome (HLHS). The mutant embryos were also mosaic, displaying a range of mutagenesis and mosaicism, together with complex CHD phenotypes (Liu et al. 2017). However, there was clear phenotypic enrichment in digenic specimens with high rates of mutagenesis, corroborated by other evidence that the two genes act synergistically to cause the HLHS phenotype. These two studies show that F0 phenotyping is feasible, although it is important to note that there are limitations to the interpretation of the results versus typical approaches to mouse mutagenesis.

### F0 modelling in adult mice

To date, there are few incidences in the literature where F0 phenotyping screens of adult animals have been utilised. This may relate to confounds associated with mosaicism as discussed above, or could reflect the restricted applicability of F0 phenotyping experiments. For example, a high degree of mosaicism in F0 animals can be visualised by targeting *Tyr* in pigmented animals and studies have shown how extensive this might be in any targeting study (Yen et al. 2014). A number of additional factors may also contribute to the difficulties of F0 screening including the type of mutation that the researcher intends to generate (missense versus null), the inherent variability of the phenotype being studied and the predictions as to how many F0 animals need to be screened to confirm that a particular mutation has an associated phenotype. The latter is particularly relevant when considering phenotypes associated with brain and behavioural function. Given the incidence of mosaicism seen in CRISPR/Cas9 studies, brain region-specific mutations are potentially highly variable. Ultimately, the investigator may want to confirm the true nature of the mutation by testing phenotypes in subsequent generations.

The use of CRISPR/Cas9 F0 screens for behavioural studies has some parallels in earlier studies in chimeric animals although mosaicism adds a level of complexity. In such an experiment, for example, Low-Zeddies and Takahashi (2001) investigated circadian behaviour in chimeric mice combining cells from wild-type LacZ-positive animals and homozygous *Clock* LacZ-negative mutants. In addition, coat pigmentation was used to illustrate the extent of chimerism in the 137 mice generated. By investigating the degree of chimerism in the suprachiasmatic nucleus (SCN) of the hypothalamus, the central pacemaker for the circadian clock, they established that all three major circadian clock parameters (period, amplitude and phase shifting) are separable based on the pattern of cells that express the *Clock* mutation. Furthermore, they established that, even when some cells in the SCN express the *Clock* mutation, the animals are behaviourally wild-type, while the converse was also true. These findings would argue that, in CRISPR/Cas9 F0 screens where behavioural phenotype is being investigated, it is important to determine the extent and/or pattern of mosaicism in the relevant brain regions of all individuals and to reflect on whether the gene in question functions in a cell autonomous or non-autonomous manner. This chimeric analysis carried out by Low-Zeddies and Takahashi would also argue that large numbers of animals should be tested prior to establishing any genotype/phenotype associations, and that negative results (animals with a WT phenotype) are difficult or impossible to interpret. In investigating the effects of Mecp2 on behavioural rhythms, Tsuchiya et al. (2015) looked at just 4 CRISPR/Cas9 targeted F0 animals while they used an additional 8 animals to investigate molecular oscillations in the SCN. Although they demonstrated significant effects on one circadian parameter (amplitude) in both sets of animals, the study of Low-Zeddies and Takahashi would suggest that further investigations of mosaicism in the SCN of a larger population of F0 animals would be warranted.

Perhaps, the most substantive behavioural study of CRISPR/Cas9 F0 animals to date has used a combination of three gRNAs to ensure efficient biallelic knockout of target genes (Sunagawa et al. 2016). The strategy was used first on the *Tyr* gene to test the efficiency of the approach and then used to validate a high-throughput sleep screen by targeting genes whose null alleles have previously been shown to affect sleep parameters (*Bmal1, Hcrt, Cry1*/*Cry2* double knockout, *Per1*/*Per2* double knockout). Having confirmed a sleep phenotype in these positive controls, the group then proceeded to screen CRISPR/Cas9 F0 knockouts of a gene family (NMDA receptor family) for sleep deficits and identified *Nr3a* as a sleep gene candidate using this approach. In testing between 2 and 15 individuals for each gene they target, the study shows that direct screening of CRISPR/Cas9 F0 individuals can successfully identify mutants, thus validating the causality of the target gene. However, questions remain as to the universal applicability of the approach, considering the degree of technical investment required for the production and characterisation of mutants, the variability in genetic outcomes, and the numbers of animals generated compared to screens where the mutation has been inherited through the germline. Ultimately, careful consideration of the costs and time required relative to “traditional” approaches is needed.

On the other hand, in particular circumstances, genetic mosaicism can be advantageous: Screening of CRISPR/Cas9 F0 individuals can provide useful additional information on the function of genes and mutant alleles. A recent study attempting to introduce a missense mutation in mouse *Eef1a2*, for example, highlights the complexity of the outcome of the technology while enabling investigators to salvage some useful information from F0 animals (Davies et al. 2017). Homozygous null mutations in mice result in early postnatal lethality while dominant de novo missense mutations in humans, including G70S, are associated with developmental delay, intellectual disability and autism. Attempts using CRISPR/Cas9 to generate animals with a single G70S mutation failed while most F0 animals carried biallelic mutations including deletions and del/G70S compound alleles. While most of these F0 mice exhibited early postnatal lethality, the findings did confirm that the G70S mutant protein was essentially non-functional. Furthermore, 6 out of the 35 F0s generated showed clear evidence of mosaicism with a clear variability in the cellular neuronal phenotypes seen in multiple brain sections and disparities between tail genotypes and *Eef1a2* expression in brain and muscle. Screens in F0 mice can also be used to investigate the effects of a mutation in specific cells or tissues and may represent a speedy alternative to using complex crosses to generate conditional mutants. This approach has been considered by Zhong et al. (2015) to investigate retinal dysfunction in mosaic F0s associated with *Kcnj13* deletion. Nevertheless, however promising these studies might be, care must be taken in interpreting the results while considering the nature and extent of the individual cellular mosaicism.

### Challenges and limitations of F0 phenotyping

From these examples, it is clear that phenotypes in F0 embryos or animals can be obtained and characterised, and that they can provide corroborating evidence for genotype–phenotype relationships. There are, however, limitations to this approach. Specifically, it is unclear if mutagenesis of genes that function in a cell autonomous or non-autonomous fashion will be equally likely to yield a phenotype. One can envision that mutating a gene for a secreted factor might display a dosage curve relative to the number of cells carrying the relevant mutation. By contrast, in the case of a gene required autonomously for cell growth, compensation by unmutagenised cells could affect the presentation of a phenotype. Unlike chimeric analysis where the cell of origin is marked, this compensation may be indistinguishable if only a few cells are mutagenised. Additionally, it is not possible to interpret the negative phenotypic presentation as the lack of phenotype could be due to incomplete editing, or compensation from unmutagenised cells as noted above. Moreover, given recent reports that variable expressivity and incomplete penetrance are a common feature of gene knockouts (Dickinson et al. 2016; Karp et al. 2017; Meehan et al. 2017), it would be difficult to establish the source of such variance in a genetically mosaic F0 embryo or animal.

As discussed above, it is evident that phenotypes from F0 mutagenesis can be observed and interpreted, while a lack of phenotype cannot. Therefore, the full realisation of the potential of F0 screening will require better methods both to determine and quantitate the alleles in each mutant. PCR and cloning-based approaches can identify the range of alleles, but cannot accurately quantitate the abundance of each allelic species. Methods employing high-throughput sequencing have been developed, but are cost prohibitive due to the use of multiple sequencing libraries (Tsai et al. 2015). Regional mosaicism (different allelic combinations in different tissues) is an additional potential caveat, but it is difficult to assess the extent to which these allelic combinations might contribute to any individual phenotype. Neither of these issues is intractable but simply requires concerted tool development effort. With future advancements, the ability to describe a dose curve of mutagenesis and relate this to phenotypic presentation could be quite powerful, providing in a single experiment an “allelic series” not available using standard approaches.

## When are CRISPR/Cas9-generated mosaics appropriate experimental models?

CRISPR/Cas9 can generate complex mosaics of unpredictable genetic composition that are difficult to fully characterise. Nevertheless, these can be attractive models for studies as illustrated above. Ultimately, the validity of such an approach depends on the specific experimental context and the scope of the interpretations reported. Because negative data cannot be reliably interpreted (all F0s without phenotypes could potentially be false negatives), F0 mutagenesis can only function as a “rule in” screen but cannot inform the role of genes in a given process for which no phenotype is observed. This is in contrast to the large-scale KO phenotypic screen of the IMPC, where the lack of phenotype in a carefully controlled context can be interpreted as an affirmative claim of wild-type function (Dickinson et al. 2016). However, an F0 screen might have significant value as a tool to rapidly screen through a large number of candidate disease genes, allowing the investigator to identify individual candidates for deeper analysis. Given the recent growth in the number of putative disease causing variants, F0 screens could provide a compromise solution that balances the need for rapid validation with the understanding that there will be a significant false-negative rate.

Although CRISPR/Cas9 methods and technologies are relatively recent, published studies already predict that the effectiveness of an F0 screen will hinge on the consideration of numerous factors. Consequently, in designing an F0 screen, we propose that one pay particular attention to (i) the choice of control, (ii) the experimental feasibility, (iii) the adherence to the Reduction and Refinement principles and (iv) the reproducibility of the study: (i) F0 founders will generally be obtained from embryos of super-ovulated donor females, further manipulated and re-implanted in pseudo-pregnant foster females, processes that are not without an impact on the biology of the offspring (Ertzeid and Storeng 2001). Will wild-type animals obtained by natural mating constitute the appropriate baseline for the experiment or must mock-mutated animals be generated alongside mutants for phenotyping? (ii) The complete genetic characterisation of F0 mutants requires a heavy investment in terms of sequencing the multiple alleles represented in the mosaic. Furthermore, fully understanding the distribution of these genotypes throughout the body may not be feasible. Definitive association of a phenotype to a genotype may require confirmation employing germline transmitted mutations. (iii) What is the impact of these experimental choices viewed from the angle of the Reduction and Refinement principles? Even though the CRISPR/Cas9 system is increasingly efficient, generating whole mutant cohorts, and perhaps controls, will likely represent a heavier burden on animal welfare (including surgical intervention) compared to the generation of small numbers of founders and breeding, particularly in the case of more complex mutations that require homologous repair. Numbers may have to be further increased to accommodate the genetic variability of the mutant founder and its impact in terms of experimental “noise”. Finally, (iv) the use of CRISPR/Cas9-generated founders as experimental models raises questions for the reproducibility of the study, as it is unlikely that genetically identical (mosaic) animals will be generated upon re-injection of the CRISPR/Cas9 reagents. Particular attention will have to be paid to the detailed description of the methods for generation and validation of mutants, phenotype range and methods for evaluating the extent of mutagenesis and its correlation to phenotypic expressivity (Kilkenny et al. 2010).

In summary, CRISPR/Cas9-generated founders can be of great value in specific cases but they may not be suitable individuals to constitute for phenotyping cohorts for many other animal studies. Genetic variability, appropriate controls, animal welfare and reproducibility of experiments, as ever, have to be considered and will be impacted when choosing to phenotype first-generation RGEN-generated mutants. The choice of phenotyping genome-edited F0s remains an unusual one and requires a justification when preferred over the study of stable and well-validated mutant lines.
